# Long-term night shift work and cardiometabolic health in healthcare workers: a review of mechanisms and precision intervention strategies

**DOI:** 10.3389/fpubh.2026.1934083

**Published:** 2026-09-03

**Authors:** Xuelan Ye, Chao Huang, Jiaxin Zhou, Zhibing Qiu, Jieying Wu, Hongmei Wu, Linfeng Mo

**Affiliations:** 1 Department of Pharmacy/Health Management, Guangzhou Huashang Vocational College, Guangzhou, China; 2 Department of Basic Medicine, Jiaying University, Meizhou, China; 3 Department of Epidemiology and Health Statistics, Guilin Medical University, Guilin, China

**Keywords:** cardiometabolic health, circadian misalignment, epigenetic modifications, healthcare workers, intervention, night shift work

## Abstract

Long-term night shift work has become one of the major risk factors for the cardiometabolic health of healthcare workers. Traditional perspectives have largely attributed this to sleep deprivation. However, recent studies have revealed that circadian misalignment itself constitutes an independent pathogenic factor. This article provides a systematic review of the epidemiological evidence, pathophysiological mechanisms, and precision intervention strategies in this field. Existing studies indicate a clear dose-response relationship between night shift exposure and cardiometabolic risk, with this association being modified by sex and age and capable of causing long-term or even irreversible health damage. Mechanistically, the desynchronization of central and peripheral circadian clocks serves as the core initiating event, which can induce neuroendocrine and autonomic imbalance. Emerging mechanisms include chronic low-grade inflammation, gut microbiota dysbiosis, and epigenetic modifications (e.g., DNA methylation changes of clock genes and human endogenous retrovirus elements). Together, they constitute a complex molecular network that underlies the pathogenic effects of night shift work. Based on these mechanisms, current intervention strategies are transitioning from one-size-fits-all recommendations to individualized approaches. These include circadian phenotype-based risk stratification, chrononutrition, timed light therapy, and precision scheduling at the organizational level. However, existing research still faces challenges including poor adherence, lack of long-term follow-up data, and insufficient multidisciplinary integration. To enable precise prevention of night-shift-related health damage in healthcare workers, future efforts should integrate digital health technologies and multidisciplinary collaboration. Full-career longitudinal cohorts should be established, incorporating multidimensional data, including chronotype, gene expression, metabolic phenotypes, and lifestyle, to build individualized risk assessment systems. This will support personalized health management and minimize the cumulative cardiometabolic damage caused by long-term night shifts.

## Graphical Abstract

**Figure:**
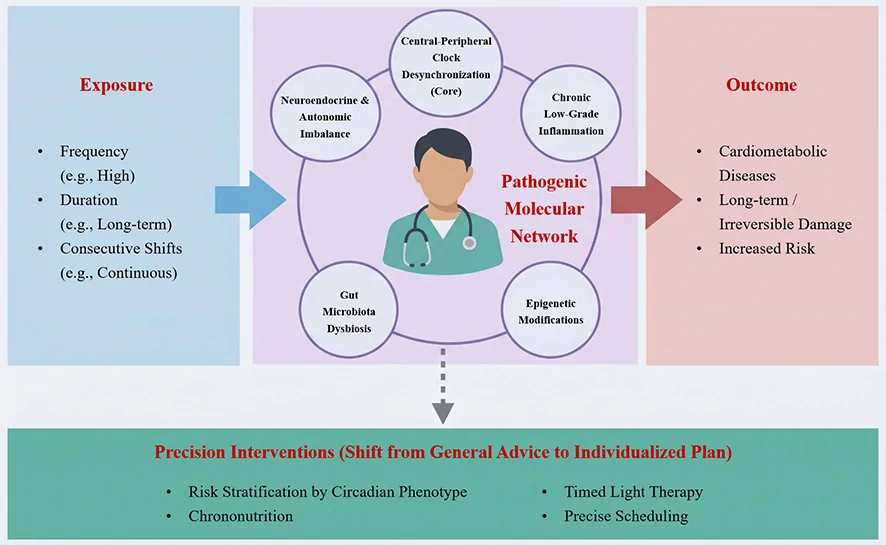

## 1 Introduction

Globally, healthcare workers are persistently engaged in high-intensity shift work schedules, with night shifts having become a professional norm. Circadian rhythm disruption is the core mechanism through which shift work affects health. Cross-sectional evidence from France and Malaysia has consistently linked night-shift work to circadian disruption among healthcare professionals. In a French cohort of 4,971 nurses, nursing assistants, and doctors, sleep timing was found to be markedly misaligned with work schedules (1). Similarly, a survey of Malaysian healthcare workers reported that 66.0% experienced poor sleep quality, with 53.4% exhibiting social jetlag, which is defined as the discrepancy in sleep midpoint between free days and workdays (2). These circadian and sleep disturbances, in turn, are associated with adverse occupational outcomes. Studies from the United States and Australia have shown that night shift work elevates end-of-day stress in healthcare workers, negatively affecting both job satisfaction and health-related quality of life (3). In China, national epidemiological data are lacking. However, multiple regional studies have consistently shown (4–9) that frontline clinical healthcare workers are often required to take consecutive night shifts. In some departments, an even more demanding continuous shift system is implemented, in which staff work for 24 h or longer with only brief rest periods. This practice further increases the risk of circadian rhythm disruption. Taken together, these findings indicate that high-frequency, long-cycle, and physiologically disruptive night shift schedules have become a common occupational challenge for healthcare workers globally.

Previous research has primarily attributed the health hazards of night shift work to sleep deprivation or reduced total sleep time. However, recent studies have provided growing evidence to the contrary. Even when sleep duration is controlled and adequate sleep is ensured, circadian misalignment alone can independently induce cardiometabolic abnormalities (10–12). For example, under simulated night shift conditions, restricting food intake to daytime alone significantly alleviated the worsening of several cardiovascular risk indicators. These included elevated blood pressure, reduced heart rate variability, and increased plasminogen activator inhibitor-1 (PAI-1) levels. This finding suggests that circadian misalignment, rather than sleep deprivation alone, is the core driver of these cardiovascular abnormalities (11). Animal experiments corroborate this, showing that chronic phase shifts inducing circadian misalignment directly lead to elevated systolic blood pressure, impaired glucose tolerance, and reduced liver mass (10). Furthermore, controlled laboratory studies have provided additional evidence. Even when sleep structure is maintained normally, healthy adults may experience mood disturbances and autonomic dysfunction after short-term circadian misalignment. Elevated levels of inflammatory markers, such as high-sensitivity C-reactive protein (hs-CRP), have also been observed (12). Collectively, these findings across different levels of investigation converge on a key conclusion: the fundamental pathological driver of cardiometabolic disease development is the desynchronization with the external environmental cycle, rather than sleep deprivation alone.

Circadian misalignment profoundly affects the cardiometabolic health of healthcare workers, making the elucidation of its mechanisms and the exploration of feasible interventions a critical priority in occupational health. Current evidence points to multiple interconnected pathways across molecular, cellular, and systemic levels. These include central and peripheral circadian clock desynchronization, hypothalamic-pituitary-adrenal (HPA) axis dysfunction, autonomic imbalance, chronic low-grade inflammation, gut microbiota dysbiosis, and epigenetic modifications (13–18). In parallel, chronobiology-based behavioral strategies, including chrononutrition (timed eating), light management, personalized shift scheduling, and supplement application, have shown preliminary promise in improving metabolic and cardiovascular parameters (11, 19–22). Nevertheless, most existing interventions remain standardized and have yet to accommodate individual variations in chronotype, sex, age, or baseline health status. Therefore, this review seeks to integrate recent findings on the epidemiological evidence, pathophysiological mechanisms, and emerging intervention strategies for circadian disruption induced by long-term night shifts. A key focus is the transition from uniform guidance to individualized precision health management. Ultimately, this paper aims to provide a scientific foundation and practical guidance for building occupational health protection systems that are specifically designed for healthcare workers.

To address the aims outlined above, this study systematically searched and screened the literature on the cardiometabolic health effects of long-term night shift work in healthcare workers. The review focused on three main dimensions: epidemiological evidence, pathophysiological mechanisms, and precision intervention strategies. The literature search was conducted in PubMed, Scopus, Science Direct, Web of Science, Google Scholar, and CNKI database from inception to June 2026. Both MeSH terms and free-text words were used as search keywords, including “night shift work,” “shift work,” “healthcare workers,” “medical staff,” “cardiometabolic health,” “cardiovascular disease,” “metabolic syndrome,” “circadian misalignment,” “circadian rhythm,” “mechanisms,” and “intervention,” along with their combinations. In addition, the reference lists of retrieved articles and relevant reviews were manually screened to identify any further studies that might have been missed. The inclusion criteria were as follows: original research, systematic reviews, or meta-analyses published in peer-reviewed journals were included. No restrictions were placed on study design. Non-English articles were considered if an English abstract was available. Conference abstracts, editorials, and opinion pieces without original data were excluded. Priority was given to studies that provided quantitative data on exposure-response relationships, mechanistic pathways, or intervention outcomes. Two authors independently screened the titles and abstracts. Disagreements were resolved through discussion or by consultation with a third author. Based on the above methodology, this article reviews the research progress on the impact of long-term night shift work on the cardiometabolic health of healthcare workers from three perspectives: epidemiological evidence, pathophysiological mechanisms, and precision intervention strategies.

## 2 Epidemiological evidence linking long-term night shift work to cardiometabolic health

To fully understand the pathophysiological disruption caused by night shift work on the cardiometabolic system, it is necessary to first establish the normal physiological patterns by which circadian rhythms regulate cardiovascular and endocrine function in healthy individuals. Under normal physiological conditions, blood pressure exhibits a circadian rhythm characterized by a morning rise, a daytime peak, and a 10%−20% nocturnal decline (dipping pattern) (23, 24). Heart rate variability (HRV) also displays circadian fluctuations, with parasympathetic tone predominating at night and sympathetic activity increasing during the day (25). At the hormonal level, cortisol peaks 30–45 min after awakening, gradually declines throughout the day, and reaches its nadir around midnight (26). Melatonin secretion begins in the dark phase of the night, typically peaks between 02:00 and 04:00, and is rapidly suppressed upon light exposure (27). The circadian oscillations of these physiological parameters are orchestrated by the suprachiasmatic nucleus (SCN). The SCN integrates light signals and transmits rhythmic instructions to downstream cardiovascular target organs. This transmission occurs via the HPA axis and the autonomic nervous system (28, 29). The fundamental harm of night shift work lies in its disruption of this precisely coordinated physiological network. This disruption is caused by inversion of the light-dark cycle and behavioral misalignment. As a result, the foundation is laid for cardiometabolic disturbances (30).

### 2.1 Dose-response associations

Determining the overall magnitude of the excess disease burden in night-shift healthcare workers compared with their day-shift colleagues is a prerequisite for systematically evaluating exposure-response relationships. A prospective cohort study from the UK Biobank, including over 280,000 workers, showed that long-term night shift workers had a 16% increased risk of atrial fibrillation (AF) (HR = 1.16) and a 22% increased risk of coronary heart disease (CHD) (HR = 1.22). Those with ≥10 years of lifetime night shifts had a 37% higher risk of CHD (HR = 1.37). Those working 3–8 night shifts per month had a 22% higher risk of AF (HR = 1.22) and a 35% higher risk of CHD (HR = 1.35). These associations showed dose-response trends. However, no significant associations were found for stroke or heart failure (31). Data from the Nurses' Health Study, which included over 189,000 registered nurses and followed them for 24 years, showed that 5–9 years and ≥10 years of rotating night shift work were associated with a 12% (HR = 1.12, 95% CI: 1.02–1.22) and 18% (HR = 1.18, 95% CI: 1.10–1.26) increased risk of CHD, respectively. The risk gradually declined after cessation of night shifts (*P* for trend < 0.001), suggesting that this effect is reversible (32). A cross-sectional study provided additional evidence. It showed that night shift workers had significantly greater carotid intima-media thickness than day workers. They also had higher levels of inflammatory markers (hs-CRP and IL-1β). Night work was independently associated with increased atherosclerosis thickness after adjusting for traditional risk factors (*B* = 2.633, 95% CI: 0.489–4.776, *P* = 0.016) (33). A prospective cohort study of Swedish healthcare employees further confirmed these findings. Fixed night workers had a 61% higher risk of ischemic heart disease (IHD) than day workers (HR = 1.61, 95% CI: 1.06–2.43). Those working more than 120 nights per year had a 53% higher risk (HR = 1.53, 95% CI: 1.05–2.21). However, no significant association with AF was observed (34).

Against the backdrop of this elevated overall risk, numerous epidemiological studies has further revealed a clear dose-response link between long-term night shifts and cardiometabolic disease risk. With respect to exposure duration, a systematic review and meta-analysis of 32,0002 participants found that each additional year of night shift work increased the risk of IHD by 0.9% (RR = 1.009, 95% CI: 1.006–1.012) (35). A second meta-analysis of 23 cohort studies confirmed this pattern, reporting that every 5-year increase in night shift duration was associated with a 7% higher risk of incident cardiovascular disease (CVD) (RR = 1.07, 95% CI: 1.04–1.09) and a 4% higher risk of CVD mortality (RR = 1.05, 95% CI: 1.03–1.06) (36). With regard to exposure frequency, a study of Taiwanese hospital employees found that a greater number of night shifts was associated with a higher risk of hypertension (adjusted OR = 1.15, 95% CI: 1.01–1.31) (37). Similarly, an investigation among female steelworkers in China revealed that those with more than 7 night shifts per month had a significantly increased risk of obesity (OR = 2.50, 95% CI: 1.17–5.35) (38). Collectively, the above evidence supports a positive dose-response relationship between night shift work and cardiometabolic risk across both dimensions of exposure intensity and duration. Table 1 summarizes the epidemiological evidence for cardiometabolic risk by exposure intensity (frequency) and duration (years).

**Table 1:** Association of exposure intensity and duration with cardiometabolic risk.

Evidence level	Exposure dimension	Study population/design	Key findings	Effect value (95% CI)	References
Background evidence (elevated overall risk)	–	UK Biobank (>280,000 workers)	Long-term night shift work was associated with a 16% increased risk of AF and a 22% increased risk of CHD. Those with ≥10 years of lifetime night shifts had a 37% higher risk of CHD.	HR = 1.16/1.22/1.37	(31)
	–	Nurses' Health Study (>189,000 registered nurses, 24-year follow-up)	5–9 years and ≥10 years of rotating night shift work were associated with 12 and 18% increased risks of CHD, respectively. Risk gradually declined after cessation of night shifts (*P* for trend < 0.001).	HR = 1.12/1.18	(32)
	–	Swedish healthcare employees (prospective cohort)	Fixed night workers had a 61% increased risk of IHD. Those working >120 nights per year had a 53% increased risk.	HR = 1.61/1.53	(34)
Dose-response evidence	Cumulative years	Meta-analysis (320,000 participants)	Each additional year of night shift, work was associated with a 0.9% increase in the risk of IHD.	RR = 1.009 (1.006–1.012)	(35)
	Cumulative years	Meta-analysis of 23 cohorts	Each 5-year increment in night shift work duration corresponded to a 7% increase in the risk of developing CVD.	RR = 1.07 (1.04–1.09)	(36)
	Cumulative years	Meta-analysis of 23 cohorts	Each 5-year increase in night shift work was associated with a 4% higher risk of CVD mortality.	RR = 1.05 (1.03–1.06)	(36)
	Night shift frequency	Hospital staff in Taiwan, China	Greater frequency of night shifts was correlated with a higher risk of hypertension.	adjusted OR = 1.15 (1.01–1.31)	(37)
	Night shift frequency	Chinese female steelworkers	Working more than 7 night shifts per month was associated with a significantly elevated risk of obesity.	OR = 2.50 (1.17–5.35)	(38)

### 2.2 Differences by sex and age

The cardiometabolic risks associated with night shift work show marked heterogeneity by sex and age. With respect to sex differences, findings from retired workers indicate that women with a history of night shift work had 3.3 times the risk of high body mass index (BMI) compared to day workers (95% CI: 1.2–10.4), whereas men predominantly exhibited an elevated risk of high triglycerides (OR = 3.9, 95% CI: 1.1–14.2) (39). In Tangshan female steelworkers, night shift duration, cumulative number of shifts, and total shift hours were all positively associated with body fat percentage and fat mass index, independent of BMI (38). By contrast, an investigation of male railway workers in southwestern China revealed that although long-term night shift work (≥10 years) was not associated with a significant increase in overall metabolic syndrome risk, it was significantly linked to systolic blood pressure (SBP) ≥130 mmHg and waist circumference ≥90 cm (OR = 1.11, 95% CI: 1.02–1.21) (40). Collectively, these sex-specific patterns suggest that night shift exposure may differentially affect metabolic phenotypes in men and women. Regarding age differences, a longitudinal study of physicians with a mean age of 30 years found that shift work significantly altered cortisol secretion rhythms. This was manifested as elevated morning cortisol levels and increased total cortisol concentrations (41), indicating that neuroendocrine stress responses already emerge in young healthcare workers. In contrast, a study based on hypertensive patients from the UK Biobank showed that long-term night shift work (particularly >10 shifts per month) was associated with an increased risk of cardiometabolic multimorbidity (HR = 1.16, 95% CI: 1.02–1.31), and this effect was more pronounced in those with morning chronotype or sleep disturbances (42). These findings suggest that age not only influences the temporal onset of night-shift-related health consequences but may also interact with other individual characteristics, such as chronotype. Notably, a study involving both current and former shift workers did not find an overall increase in metabolic syndrome risk. However, it did suggest a potential interaction between shift work and sex (43). Table 2 summarizes the sex- and age-related differences in cardiometabolic risk associated with night shift work.

**Table 2:** Sex and age differences in night shift-related cardiometabolic risk.

Stratification	Population	Key findings	Effect value (95% CI)	References
Sex	Female retirees	The risk of high BMI was 3.3 times that of the day-worker control group.	OR = 3.3 (1.2–10.4)	(39)
	Male retirees	The risk of high triglycerides was 3.9 times that of the day-worker control group.	OR = 3.9 (1.1–14.2)	(39)
	Tangshan female steelworkers, China	Both the duration and frequency of night shift work were positively associated with body fat percentage and fat mass index.	Remained significant after adjustment for BMI.	(38)
	Southwest Chinese male railway workers (≥10 years night work)	An increased risk of SBP ≥130 mmHg and waist circumference ≥90 cm.	OR = 1.11 (1.02–1.21)	(40)
Age	Physicians with a mean age of 30 years	Disruption of cortisol secretory rhythm, manifesting as elevated morning cortisol levels and increased overall cortisol concentrations.	Neuroendocrine stress reaction.	(41)
	Middle-aged and older hypertensive patients from the UK Biobank	A frequency of more than 10 night shifts per month was associated with an elevated risk of cardiometabolic multimorbidity.	HR = 1.16 (1.02–1.31)	(42)
Interaction	Current/former night shift workers	A potential interaction may exist between shift work and sex.	No overall increase in metabolic syndrome risk was observed.	(43)

### 2.3 Legacy effect

The health impairments associated with night shift work exhibit a substantial legacy effect. Even after exposure has ceased, the body's recovery from cardiometabolic damage may take an extended period. In some cases, the damage may be irreversible (31, 44, 45). Multifaceted evidence collectively confirms the objective existence of the legacy effects of night shift exposure. In terms of clinical metabolism, a comparative study was conducted on retired night shift workers and retired day workers. After adjusting for confounding factors, the study found no significant difference in the overall prevalence of metabolic syndrome between the two groups. However, retired night shift workers still exhibited specific metabolic abnormalities. These included higher BMI in women and higher triglyceride levels in men (39). This suggests that the legacy effects of night work may appear not as full metabolic syndrome, but as specific metabolic trait abnormalities. At the level of long-term survival, a significant association was found among workers with pre-existing cardiometabolic diseases. Those with a history of long-term night shift exposure had a significantly higher mortality rate (46). Compared with individuals without night shift exposure, the risk of all-cause mortality was increased by 28% (HR = 1.28, 95% CI: 1.02–1.62). The risk of cardiometabolic mortality was increased by 57% (HR = 1.57, 95% CI: 1.01–2.42), and the risk of cardiovascular mortality was increased by 61% (HR = 1.61, 95% CI: 1.02–2.53) (46). These findings indicate that the detrimental effects of night shift work can persist beyond retirement and further worsen the prognosis of those already affected by disease. Mechanistically, sleep deficiency and circadian misalignment are considered key drivers of the legacy effect. Shift work-related sleep loss can increase sympathetic tone, inflammation, glucose intolerance, and appetite hormone changes (47). When combined with sleep apnea, circadian misalignment may worsen cardiometabolic risk via autonomic dysfunction (47). These observations suggest that sleep-related comorbidities may act as important effect modifiers of the legacy phenomenon. Taken together, the adverse cardiometabolic consequences of night shift work are both prolonged and partially irreversible, underscoring the necessity of early preventive measures and ongoing health monitoring. Table 3 summarizes the epidemiological evidence for night shift exposure and cardiometabolic legacy effects.

**Table 3:** Epidemiological evidence on night shift exposure and cardiometabolic legacy effects.

Stratification	Population/study design	Key findings	Effect value (95% CI)	References
Clinical metabolic outcomes	Retired night vs. day workers	1. Women exhibited higher BMI. 2. Men showed higher triglyceride levels. 3. These findings suggest that abnormalities in specific metabolic phenotypes may persist over time.	No significant difference in overall metabolic syndrome risk.	(39)
Long-term survival outcomes	Workers with diagnosed cardiometabolic disease	A history of long-term night shift work was linked to elevated all-cause mortality.	HR = 1.28 (1.02–1.62)	(46)
		A history of long-term night shift work was linked to elevated cardiometabolic disease mortality.	HR = 1.57 (1.01–2.42)	
		A history of long-term night shift work was linked to elevated cardiovascular disease mortality.	HR = 1.61 (1.02–2.53)	
Mechanistic explanation	Review	Sleep insufficiency is associated with elevated sympathetic activity, enhanced inflammatory responses, impaired glucose tolerance, and dysregulation of appetite-related hormones.	A key contributor to the legacy effect	(47)
		In the presence of comorbid obstructive sleep apnea, autonomic nervous system dysfunction may further potentiate cardiometabolic risk.	Sleep-related comorbidities may act as effect modifiers.	

## 3 Pathophysiological mechanisms: from rhythm disruption to target organ injury

**Figure 1:**
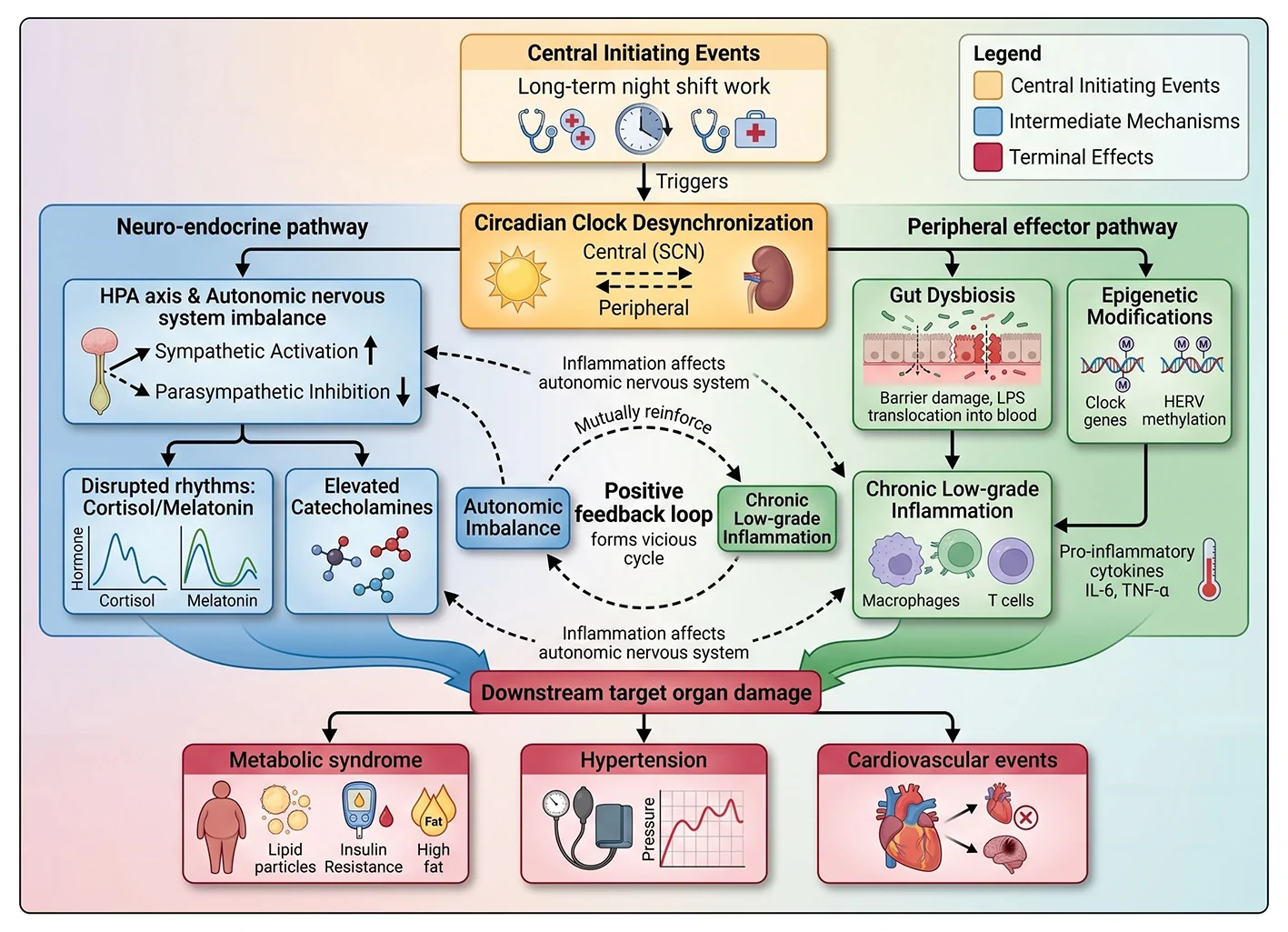
Core mechanistic network of cardiometabolic injury induced by long-term night shift work in healthcare workers. Long-term night shift work among healthcare workers, through the core trigger of circadian desynchronization, simultaneously activates the neuro-endocrine pathway and the peripheral effector pathway. These two pathways amplify the damage through a vicious positive feedback loop, ultimately leading to metabolic syndrome, hypertension, and even serious cardiovascular events.

In healthcare workers, long-term night shift work acts through the core trigger of circadian desynchronization. This simultaneously activates two pathways: the neuro-endocrine pathway and the peripheral effector pathway. These two pathways amplify the damage through a vicious positive feedback loop, ultimately leading to metabolic syndrome, hypertension, and even serious cardiovascular events. Figure 1 illustrates the core mechanistic network of cardiometabolic damage induced by long-term night shift work in healthcare workers.

### 3.1 Desynchronization of central and peripheral circadian clocks

Long-term night shifts desynchronize the internal circadian rhythm from the external light-dark cycle, leading to central-peripheral clock misalignment (Figure 1) (48). The SCN serves as the master pacemaker of the central circadian clock. It is primarily regulated by light signals. In contrast, the circadian rhythms of peripheral tissues, including the liver, adipose tissue, and heart, are more responsive to non-photic cues such as feeding schedules, temperature oscillations, and hormonal fluctuations (49–51). Under physiological conditions, the central and peripheral circadian clocks remain highly synchronized, working together to maintain internal homeostasis. However, night shift work disrupts this coordination. When night workers are exposed to artificial light and eat during nighttime hours, the central SCN continues to maintain a daytime rhythm. At the same time, peripheral organs shift their phases due to changes in behavioral patterns. This leads to internal circadian disruption (52). This uncoupling between central and peripheral clocks is regarded as the key initiating step in night shift-related metabolic damage. At the molecular level, healthcare workers with long-term rotating night shifts show marked changes in core clock gene expression. Specifically, CLOCK, NPAS2, PER1, PER3, and REV-ERBα are upregulated, while BMAL1 and CRY1 are downregulated. This gene expression imbalance is closely correlated with higher postprandial triglyceride levels and insulin resistance (53). An intervention study in shift workers found that 12 weeks of intermittent light therapy had no significant effect on metabolic or inflammatory markers. However, it did significantly reduce REV-ERBα expression in peripheral blood mononuclear cells and increase the REV-ERBα/BMAL1 ratio (54). These results indicate that peripheral clocks are somewhat plastic and responsive to light, offering experimental support for the molecular mechanisms of night shift-related circadian disruption. Furthermore, the effects of central-peripheral circadian uncoupling are not confined to metabolic organs. They may also involve the immune and barrier systems. Animal studies have shown that circadian disruption can disturb the normal rhythmic expression of clock genes such as Bmal1 and Per2 in intestinal epithelial cells, impair gut barrier function, and thereby exacerbate inflammatory responses (55). These observations broaden the pathophysiological relevance of circadian desynchronization, indicating its widespread role in systemic inflammation and metabolic disturbances. Furthermore, one important point should be noted. Light is a core zeitgeber. When its biological effects are analyzed, a clear distinction must be made between general illumination and spectral composition. The circadian regulatory effects of biologically active light are not determined by intensity or exposure duration alone. They are critically dependent on another factor. This factor is the stimulation of short-wavelength blue light (approximately 460–480 nm) on intrinsically photosensitive retinal ganglion cells (ipRGCs). These ipRGCs express melanopsin. Through this expression, they transmit non-image-forming visual signals to the SCN. Their sensitivity to short-wavelength light is significantly enhanced at night (56–58). This mechanism has particular occupational relevance for healthcare workers. Healthcare workers are exposed to screen-based blue light during night shifts. This exposure is unavoidable. It occurs at a time that contradicts the physiological nocturnal rhythm. It can interfere with SCN pacemaking via the non-image-forming visual pathway. This interference exacerbates the desynchronization of central and peripheral circadian clocks (59). Therefore, light intervention strategies should be developed carefully. These strategies should regulate light intensity and exposure timing. In addition, spectral filtering should be incorporated. An example is blue-light blocking. Phase-specific light design should also be included. These measures will help achieve more precise circadian regulation.

### 3.2 Dysregulation of the neuroendocrine–autonomic nervous system axis

Circadian disruption can directly interfere with the balance of the HPA axis and the sympathetic-parasympathetic nervous system, leading to neuroendocrine and autonomic dysfunction (Figure 1) (60–62). These two systems act in concert to preserve cardiometabolic stability, and their dysregulation constitutes a key mechanism in the pathogenesis of night shift-related health impairments. Night shift work is often associated with delayed or inhibited melatonin secretion and altered cortisol rhythms. These changes negatively affect glucose metabolism, lipid balance, and blood pressure regulation (63). The local circadian clock in the adrenal gland, together with the central SCN, coordinates the rhythmic secretion of glucocorticoids (GCs). Circadian misalignment caused by night shifts can disrupt the normal diurnal oscillations of GCs, thereby exerting widespread effects on metabolism, cardiovascular function, and inflammatory responses (64). Research has shown that blunted nocturnal blood pressure dipping is common among night shift workers. This abnormal pattern is considered a potential indicator of adverse cardiovascular prognosis and is closely associated with sleep deprivation (65). Furthermore, a randomized controlled trial found that timed light therapy significantly improved the diurnal blood pressure rhythm in night shift workers, increased the proportion of normal dippers, and enhanced glucose tolerance. These benefits were associated with lower plasma metanephrine and norepinephrine, indicating that sympathetic overactivity plays a key role in night shift-related cardiometabolic injury (66). Collectively, these findings suggest that the neuroendocrine and autonomic systems do not work in isolation. Instead, they interact through a complex network to mediate night shift-induced cardiometabolic damage. HPA axis overactivation increases sympathetic tone, and autonomic imbalance can reciprocally affect hormone rhythms, creating a vicious cycle that amplifies overall cardiometabolic risk. Furthermore, it should be noted that when analyzing non-photic stressors in the healthcare occupational environment, occupational noise represents an important and non-negligible factor. Environmental noise (e.g., monitor alarms, ventilator operation, infusion pump alerts, telephone rings, and staff conversations) is common in intensive care units, emergency departments, and operating rooms. The equivalent sound levels often exceed the WHO-recommended nighttime limit of 40 dB LAeq, sometimes reaching above 70 dB (67–69). Noise exposure can be repeated or sustained. Such exposure can activate the amygdala and hypothalamic paraventricular nucleus via auditory pathways. This activation triggers stress responses in the HPA axis and the sympathetic-adrenal-medullary axis. This leads to disrupted cortisol secretion rhythms and elevated catecholamine levels (70). Chronic exposure to this stress state can induce insulin resistance, visceral fat accumulation, and hypertension, ultimately promoting atherosclerosis (71). It is important to emphasize that the physiological effects of noise exposure and circadian misalignment may produce synergistic amplification. During night shifts, healthcare workers are in a state of heightened stress sensitivity due to sleep deprivation and circadian disruption (41, 72). Noise acts as an additional stressor. It can further exacerbate HPA axis dysfunction and cortisol rhythm disruption. This amplifies the adverse cardiometabolic effects of night work (71, 73, 74). Therefore, noise should be considered an important occupational cardiometabolic risk factor. It is independent of circadian disruption. However, it closely interacts with circadian disruption.

### 3.3 Chronic low-grade inflammatory response

Recent studies have revealed that long-term night shift work promotes cardiometabolic damage not only through central-peripheral circadian desynchronization and neuroendocrine rhythm disruption (e.g., impaired adaptation of melatonin and cortisol rhythms to night work) (48), but also through emerging mechanisms including chronic low-grade inflammation (75, 76), gut microbiota dysbiosis (77), and epigenetic alterations (78) (Figure 1). These mechanisms are interwoven and collectively form a complex network underlying the pathogenic effects of night shift work. Multiple population-based studies have confirmed that inflammatory markers are significantly elevated in night shift workers. For instance, male night-shift medical staff had notably higher neutrophil-to-lymphocyte ratio (NLR) and C-reactive protein-to-albumin ratio (CAR) compared to day-shift workers (NLR: 2.19 vs. 1.84, *P* = 0.012; CAR: 0.44 vs. 0.24, *P* = 0.002). These findings suggest a systemic low-grade inflammatory state (79). In addition, a large analysis using NHANES data found that weakened rest-activity rhythms were significantly correlated with higher white blood cell counts, neutrophil counts, and systemic immune-inflammation index (SII) (80). Moreover, elevated whole-blood IL-2 and reduced IL-10 levels were observed in night shift workers, indicative of a net pro-inflammatory shift in the cytokine balance (81). Meanwhile, a study of emergency physicians provided complementary transcriptional evidence. Gene expression analysis of whole blood after night shifts showed activation of Toll-like receptor and innate immune pathways (82). This further supports the role of innate immunity and inflammation in night shift-related pathology. Taken together, these observations show that night shift exposure can activate the innate immune system through multiple mechanisms, creating a sustained low-grade inflammatory environment. Over time, this chronic inflammation may contribute to insulin resistance, lipid disorders, and endothelial dysfunction, ultimately raising the risk of cardiometabolic disease (Figure 1).

### 3.4 Intestinal microecological dysregulation

The gut microbiota acts as a crucial link between circadian rhythms and metabolic health. However, its own rhythmic patterns are also disturbed by night shift work. Animal studies have shown that disruption of circadian rhythms or deletion of Per1/2 genes can damage the intestinal epithelial barrier. This increases gut permeability, allowing lipopolysaccharide (LPS) to enter the bloodstream and trigger systemic inflammation (Figure 1) (83). Notably, certain gut microbial metabolites play a protective role in circadian regulation. Urolithic acid (UA), for example, can improve gut barrier function and influence central SCN rhythms by regulating clock gene expression in intestinal epithelial cells. This points to a possible role of the gut–microbiota–brain–heart axis in night shift-related disorders (55). Furthermore, in a high-fat diet-induced murine model, supplementation with edible bird's nest (EBN) was shown to modulate microbial community structure, decrease LPS biosynthesis, preserve intestinal barrier integrity, and improve hepatic–intestinal axis function, along with upregulation of hepatic circadian pathways, ultimately attenuating insulin resistance and inflammatory responses (84). These findings provide a theoretical basis for intervention strategies targeting the gut microbiota.

### 3.5 Epigenetic modifications

Beyond the mechanisms described above, epigenetic modifications have also shown potential roles in the pathogenesis of night shift-related effects. Transcriptomic analysis of whole blood collected after night shifts has shown that hundreds of genes are significantly altered in their expression. These genes are involved in immune function, metabolism, and stress response pathways (82). This suggests that circadian misalignment may reprogram gene expression via DNA methylation, histone modifications, and related epigenetic mechanisms (Figure 1). Existing evidence indicates that dysfunction of clock genes (e.g., BMAL1) can influence the epigenetic control of downstream genes involved in metabolism and inflammation. This may contribute to myocardial damage and metabolic disturbances (85). More direct evidence comes from a study of night shift workers, which demonstrated that these individuals show changes in the methylation levels of several human endogenous retrovirus elements and immune- or inflammation-related genes. This suggests that methylation of human endogenous retroviruses could be used as a potential epigenetic biomarker to monitor the biological effects of night shift exposure (86). Furthermore, night shift work may contribute to cardiometabolic disease and cancer risk by altering DNA methylation of clock genes and disrupting the circadian regulatory network (87). In female hospital staff, the links between clock gene methylation (e.g., CRY1, CSNK1A1, CSNK1D, CSNK1E, DEC1, RORA) and obesity or lipid levels varied by night work status, suggesting that night shift work may modulate susceptibility to cardiometabolic risk through epigenetic alterations of clock genes (88). However, despite initial evidence pointing to night shift-related epigenetic alterations, there is still a lack of longitudinal epigenetic studies in night shift populations. As a result, the causal links and long-term consequences remain to be established.

In conclusion, chronic inflammation, gut dysbiosis, and epigenetic modifications form a complex network that mediates night shift-related cardiometabolic injury. This network offers multiple potential targets for precision intervention strategies.

## 4 Precision intervention strategies: from general recommendations to individualized approaches

Table 4 summarizes the strategies, evidence, and challenges across different intervention levels.

**Table 4:** Summary of precision intervention strategies.

Intervention domain	Core strategy	Key evidence and outcomes	Quality of evidence/ feasibility	Implementation points and challenges	References
Risk Stratification	Building a multidimensional risk assessment framework	1. Evening chronotypes sustain less sleep loss on night shifts but present elevated risks for visceral adiposity, blood pressure, pulse rate, and LDL-C. 2. Morning chronotypes differ significantly only in LDL-C levels. 3. Both shift frequency and cumulative night work duration show dose-response associations with fasting glucose, BMI, TC, LDL-C, and blood pressure. 4. Sleep quality serves as a significant mediator in these relationships.	1. Evidence: Moderate (mainly cross-sectional; cohort data growing). 2. Difficulty: Moderate–high (needs multi-source data and ML model development).	1. Incorporate chronotype, shift-related factors, sleep quality, metabolic parameters, and psychosocial determinants into the assessment framework. 2. Build upon existing cohort resources such as the 1001 Nights and Klokwerk+ studies. 3. Apply machine learning approaches in future work to construct personalized risk prediction models.	(89–96)
Chrononutrition Strategies	Meal timing adjustment for circadian alignment	1. Nighttime eating delays the postprandial triglyceride peak to 19:30 and glucose/insulin peaks to 23:30. 2. Daytime food consumption induces a more pronounced pro-inflammatory response than nighttime eating, challenging the traditional notion that nighttime eating is inherently detrimental. 3. A six-month dietary intervention yielded a significant reduction in LDL cholesterol.	1. Evidence: Low–moderate (mostly small, short-term trials). 2. Difficulty: High (hard to implement in busy clinical settings; adherence is poor).	1. The appropriate eating window needs to be redefined. 2. Combine caloric restriction with shifting main energy intake to the active phase of the circadian cycle. 3. Evaluate feasibility and tolerability outcomes, including hunger, sleepiness, and occupational performance. 4. Evidence-based guidelines are currently lacking, and implementation barriers are common.	(97–100)
Light and Sleep Management	Timed light therapy + strategic napping + sleep hygiene education	1. A 12-week light therapy regimen increased the prevalence of dipping blood pressure from 29% to 58%, improved glucose tolerance by 22%, and was correlated with reduced norepinephrine levels. 2. Light exposure downregulated REV-ERBα expression and elevated the REV-ERBα/BMAL1 ratio, with molecular circadian changes emerging before clinical phenotypic shifts. 3. Brief naps during night shifts mitigated adverse blood pressure responses and facilitated restoration of normal nocturnal dipping profiles.	1. Evidence: Moderate (RCTs exist but small). 2. Difficulty: Moderate (needs equipment and personalized design).	1. Light intensity, wavelength, and duration should be individually tailored. 2. Nighttime noise control and measures to enhance sleep continuity are essential. 3. Restoration of molecular circadian rhythms may serve as an early indicator of phototherapy responsiveness.	(54, 65, 66, 101)
Precision Use of Pharmaceuticals and Supplements	Pharmacological agents + smoking cessation + vitamin D tracking	1. The melatonin pathway is considered a key mechanism, but clinical evidence remains insufficient. 2. Night shift work activates inflammatory pathways such as Toll-like receptor signaling, and the resulting gene expression profile resembles that of major depressive disorder. 3. Night-shift healthcare workers have a higher smoking rate (OR = 2.10). 4. Vitamin D has been identified as a key monitoring indicator in the Klokwerk+ study.	1. Evidence: Low (mostly mechanistic; no RCTs). 2. Difficulty: High (needs biomarkers; concerns over individual variation and safety).	1. Implementation should be guided by individual risk stratification, avoiding uniform protocols. 2. Biomarker-directed, targeted intervention studies are required. 3. Smoking cessation programs in the workplace should be integrated into holistic occupational health management.	(82, 96, 102, 103)
Precision Scheduling in Organizational Management	Individualized scheduling + principle of minimal circadian disruption + supportive measures	1. High shift frequency, consecutive night shifts, and three-shift rotation are strongly associated with sleep disruption and metabolic impairment. 2. The 1,001 Nights cohort observed the poorest health profiles in both long-term night workers and those without any night shift exposure, suggesting that frequent transitions between shift types may impose greater circadian disruption than stable night schedules. 3. Evening chronotypes are better adapted to night work, whereas morning chronotypes should generally avoid night shifts.	1. Evidence: Moderate (strong observational data, few intervention studies). 2. Difficulty: High (needs collaboration among management, health teams, and staff; requires changing established practices).	1. Prioritize forward-rotating schedules (e.g., morning to evening to night). 2. Maintain the same shift type for at least 7 consecutive days; limit consecutive night shifts to a maximum of 3–4 days; provide sufficient rest periods between shift blocks. 3. Integrate voluntary choice with chronotype-based assignment, periodic health surveillance, sleep specialist consultation, and tailored dietary advice. 4. Complementary supports: on-site nutritional counseling, fitness facilities, psychosocial resources, routine medical examinations, and continuous monitoring through electronic health records.	(45, 90, 95, 104, 105, 111)

Evidence level: (1) High: multiple RCTs or high-quality meta-analyses; (2) Moderate: limited RCTs, good cohort/case-control studies; (3) Low: cross-sectional, mechanistic, or expert opinion.

Difficulty: (1) Low: easy, low-cost, good adherence; (2) Moderate: needs resources and personalization; (3) High: requires systemic change, multi-team work, or faces major adherence issues.

### 4.1 Risk stratification: from group data to personal assessment

Precision intervention begins with systematic risk assessment of night-shift healthcare workers to identify those at highest risk. Current evidence shows that the cardiometabolic effects of night shift work vary considerably between individuals. This variability is shaped by factors such as chronotype, sex, age, cumulative exposure, and lifestyle (89–93). Consequently, building a multidimensional risk stratification model is a necessary first step for implementing precision intervention. Notably, different circadian phenotypes exhibit marked differences in adaptability to night shifts and their associated risk profiles. For example, eveningness individuals appear to sustain less sleep loss during extended night shift schedules (90). However, when compared to morningness individuals, eveningness shift workers have higher risks for visceral fat, SBP, pulse rate, and low-density lipoprotein cholesterol (LDL-C) (89). In contrast, morningness individuals only show differences in LDL-C levels. These findings suggest that risk stratification based on circadian phenotype is not only feasible but also provides a critical entry point for subsequent personalized interventions. Beyond chronotype, shift-related factors, including shift frequency and cumulative night work duration, are also critical determinants of risk accumulation. These factors have been consistently linked in a dose-response manner to a range of metabolic disturbances, such as elevated fasting glucose, increased BMI, raised total cholesterol (TC) and LDL-C, and higher blood pressure (91, 93). Notably, sleep quality plays a significant mediating role in these associations (91, 94). Therefore, an effective risk stratification model should incorporate a broad range of factors, including chronotype, shift patterns, sleep quality, metabolic markers, and psychosocial variables. Currently, cohort studies (e.g., the 1,001 Nights cohort (95), the Klokwerk+ study (96)) have provided high-quality data foundations for model development and validation through the use of questionnaires, biomarkers, activity monitoring, and database linkage. In the future, machine learning methods may be used to build personalized risk prediction models. These tools could offer a more evidence-based approach to allocating resources for precision interventions. Ultimately, this would support the targeted management of individuals at elevated risk.

### 4.2 Chrononutrition strategies

Chrononutrition focuses on aligning meal times with the endogenous circadian clock. It is regarded as a promising approach to managing metabolic problems in night shift workers. However, the circadian misalignment inherent to night shift work makes both the timing and composition of food intake equally critical in pathological intervention. Research shows that meal patterns during night shifts have a substantial impact on metabolic outcomes. For instance, eating during night shifts resulted in a triglyceride peak at 19:30, while the glucose and insulin area-under-the-curve peaks were delayed until 23:30 (97). In addition, greater total food intake on workdays was significantly associated with higher lipid levels among night-shift nurses (98). More importantly, the pro-inflammatory effect of food intake during the daytime was greater than that of nighttime eating, regardless of shift type or work status (98). This challenges the conventional belief that nighttime eating is inherently harmful. Instead, it suggests that the determination of an optimal eating window should take individual differences in endogenous circadian rhythms into full account. Mechanistically, a preliminary study found that among rotating night shift workers, both the postprandial triglyceride area under the curve (PPTG AUC) and its peak value correlated positively with fasting insulin and Homeostatic Model Assessment of Insulin Resistance (HOMA-IR). This provides initial evidence that mistimed eating may directly impair insulin sensitivity (99). In terms of intervention, although long-term nutritional programs had limited success in weight control, a 6-month intervention did significantly reduce LDL-C (97). Overall, research on nutritional interventions for night shift workers remains limited. Evidence-based guidelines are lacking, and participants frequently report adherence difficulties (100). Given the mechanistic insights and preliminary data, future randomized controlled trials should systematically test the effects of caloric restriction and meal timing adjustments (e.g., shifting main calorie intake to the biological daytime) on cardiometabolic outcomes. At the same time, feasibility and tolerability measures, including hunger, drowsiness, and job performance, should be assessed (97). Such efforts are essential to establish a solid evidence base for personalized and feasible chrononutritional approaches.

### 4.3 Light and sleep management

Light serves as the most powerful zeitgeber for the mammalian circadian system, and strategic light exposure has been shown to mitigate some of the circadian disruption associated with night shift work. In a randomized controlled trial (RCT), 12 weeks of scheduled light therapy significantly improved both ambulatory blood pressure profiles and glucose tolerance in rotating night shift employees. Specifically, the prevalence of the dipping blood pressure pattern increased from 29 to 58%, and plasma glucose levels during oral glucose tolerance testing declined by 22% (*P* < 0.05). These benefits were linked to lower levels of norepinephrine breakdown products (66). However, another intervention using warm white light (10,000 lx, 30 min daily) did not improve sleep or metabolic parameters, but it did significantly reduce REV-ERBα gene expression in peripheral blood mononuclear cells and increase the REV-ERBα/BMAL1 ratio. These changes persisting for 12 days after the intervention ended (54). This suggests that the regulatory effect of light on molecular rhythms may precede clinical phenotypic changes. Therefore, molecular rhythm remodeling could be used as an early biomarker to assess the effectiveness of light therapy. Regarding sleep optimization, short naps during night shifts have been found to reduce the negative impact on blood pressure. They may also help restore the normal nighttime blood pressure dip, at least temporarily, which could lower CVD risk (65). Moreover, nighttime noise and shift work can disrupt melatonin rhythms and cause sleep fragmentation. This includes longer time to fall asleep, irregular sleep patterns, and more frequent awakenings (101). Thus, maintaining sleep continuity are essential. In practice, light-based interventions should be tailored to individual shift patterns, with careful consideration of light intensity, wavelength, and timing. It should also be integrated with sleep hygiene education and strategic napping to jointly enhance circadian alignment and metabolic balance.

### 4.4 Precision use of pharmaceuticals and supplements

Currently, there are no standardized pharmacological intervention protocols specifically targeting the cardiometabolic risks of night shift workers. However, mechanistic studies have provided some initial clues for precision pharmacotherapy. Melatonin and its receptor-mediated pathways involved in oxidative stress, inflammation, and vasoconstriction are considered key nodes in the pathogenesis of night shift-related health effects (102). Theoretically, exogenous melatonin may offer protective benefits, but relevant clinical evidence remains lacking. Of note, night shift work can activate innate immune and inflammatory pathways, such as Toll-like receptor signaling. In fact, gene expression profiles in blood after night shifts closely resemble those seen in major depressive disorder (82). This raises the possibility that anti-inflammatory or neuromodulatory drugs could be useful in certain subgroups. Separately, a cohort study in Singapore reported that night-shift healthcare workers had a much higher rate of smoking (OR = 2.10) (103). Since smoking is a modifiable risk factor for cardiovascular disease, workplace smoking cessation programs should be part of a broader management plan. Regarding supplements, vitamin D levels are closely related to light exposure and immune function, and have been identified as a key monitoring indicator in the Klokwerk+ study (96). However, the efficacy of vitamin D supplementation still awaits validation through large-scale clinical studies. In general, the use of medications and supplements must be based on individual risk stratification, avoiding one-size-fits-all approaches. Looking ahead, biomarker-guided targeted intervention trials (e.g., the use of specific anti-inflammatory agents in individuals with heightened inflammatory status) will be needed to advance the development of precision pharmacological strategies.

### 4.5 Precision scheduling in organizational management

Organizational-level scheduling is a key determinant of how night work affects health. The design of shift schedules directly shapes the extent to which healthcare workers experience cumulative circadian disruption. Epidemiological evidence consistently shows that high shift frequency, prolonged sequences of consecutive night shifts, and three-shift rotation systems are strongly associated with decreased sleep quality and metabolic health impairment (45, 104). Interestingly, the 1,001 Nights cohort study found that both long-term night shift workers and those who never worked night shifts had the poorest health outcomes (95). This suggests that frequently changing between different shift types may cause greater circadian disruption than consistently working fixed night shifts. Based on these findings, scheduling should move from a reactive approach to a proactive one. In practice, night and rotating shifts should be arranged through a combination of voluntary participation and personalized scheduling. Assignment should consider each worker's chronotype. Additional supportive measures, such as routine health checks, temporary reassignment to day shifts when needed, access to sleep specialists, and tailored nutritional advice, should also be provided to help reduce health risks (45). Furthermore, at the operational level, precision shift scheduling should be guided by the principle of minimizing circadian disruption. This entails a preference for forward-rotating schedules (e.g., morning to evening to night), extended durations of identical shift blocks (ideally no fewer than 7 days), restriction of consecutive night shifts to a maximum of 3–4 days, and the provision of adequate rest intervals between shift blocks (104). At the same time, individual chronotype should be a key consideration in scheduling decisions. Evening types tend to adapt better to night work, while morning types should generally be assigned to day shifts whenever possible (90, 105). However, the practical implementation of the above precision scheduling principles faces severe human resource constraints. Healthcare institutions worldwide face nursing shortages (106–108). Staffing is already limited in this context. If shifts are strictly matched based on morningness and eveningness chronotypes, the pool of available night workers will shrink. This may further exacerbate shift density or prolong consecutive night shifts, thereby inadvertently increasing health risks. Therefore, the implementation of precision scheduling requires a dynamic balance between circadian protection principles and staffing realities. Binding constraints should be established, such as minimum staffing levels and maximum consecutive night shifts. A feasible implementation plan should be developed. This plan requires collaboration among several parties. These parties include hospital management, nursing departments, unions, and occupational health services. It is worth noting that schedule optimization is only one component of a comprehensive intervention package and cannot resolve all issues in isolation. When staffing constraints cannot be immediately relieved, compensatory protective measures should be prioritized (e.g., pre-shift napping opportunities and psychological stress support channels). Such measures can compensate for scheduling limitations through a “combined intervention” model (109, 110). At the same time, organizational-level support measures should be provided in parallel. These encompass on-site nutritional guidance, exercise facilities, psychosocial resources, and regular health examinations (111). In parallel, a dynamic monitoring system based on electronic health records should also be set up to track changes in metabolic markers over time. Ultimately, precision scheduling is not merely an optimization of time arrangements, but a systematic project that integrates individual biological characteristics, job demands, and health management. Its success depends on the joint involvement of hospital leadership, occupational health teams, and the workers themselves.

## 5 Discussion

This article has reviewed the epidemiological evidence, pathophysiological mechanisms, and precision intervention strategies concerning the cardiometabolic health effects of long-term night shift work in healthcare workers. The available literature provides several key findings. A clear dose-response relationship exists between night shift exposure and cardiometabolic risk (35, 37). This effect shows sex-specific phenotypic differences. It also has legacy effects that persist after retirement (39, 46). Circadian misalignment is the core pathological driver. Sleep deprivation alone does not explain the effects. Central-peripheral circadian clock desynchronization can trigger a multi-tiered molecular network. This network includes neuroendocrine imbalance, autonomic dysfunction, chronic inflammation, gut microbiota dysbiosis, and epigenetic modifications (48, 75–78). The spectral characteristics of light and occupational noise exposure further enrich this mechanistic framework as important modulating factors (59, 70). Intervention strategies are changing. They are moving from general recommendations to individualized approaches. These include risk stratification, chrononutrition, timed light therapy, and precision scheduling.

Several molecular pathways have been identified. They are involved in night shift-related pathogenesis (48, 60, 61, 75, 82, 83). However, a systematic understanding is still lacking. It is unclear how these pathways interconnect. It is also unclear how they synergistically drive cardiometabolic damage. Several questions remain about the underlying mechanisms. One key question concerns inflammation. Is it a direct consequence of circadian desynchronization? Or is it a secondary event following gut barrier impairment? Longitudinal studies are needed to clarify this issue. Epigenetic modifications are another important area. They are considered a bridge. This bridge links long-term exposure to long-term health damage. However, their causal direction is unclear. Their degree of reversibility also remains to be verified. Furthermore, there remains considerable uncertainty regarding the parametric effects of different wavelengths, intensities, and timing of light exposure on SCN phase resetting (56–58). This makes it difficult to develop standardized light therapy protocols. At the intervention level, most evidence derives from short-term trials under strictly controlled conditions (66, 95–97). Whether these effect sizes can be sustained within the daily work rhythms of night shift healthcare workers remains unverified in real-world clinical settings. The synergistic effects among chrononutrition, light therapy, and scheduling optimization have also not been systematically explored.

This review has several limitations. First, the included literature is predominantly cross-sectional and short-term cohorts. Long-term prospective evidence is relatively scarce. This limits our ability to infer causality and assess long-term effects. Second, there is considerable heterogeneity across studies in night shift exposure assessment (e.g., definitions of cumulative years and frequency) and outcome measures (e.g., diagnostic criteria for metabolic syndrome). This heterogeneity complicates the integration of findings. Third, our discussion of macro-level determinants is relatively limited. These determinants include organizational management policies and occupational culture. Finally, the literature search was primarily restricted to English-language publications, which may introduce language bias.

## 6 Challenges and future perspectives

Based on the above discussion, precision health management for night-shift healthcare workers faces three core challenges that urgently need to be addressed in future research. Table 5 summarizes the limitations of current research and future directions for action.

**Table 5:** Summary of current research challenges and proposed future action framework.

Challenge	Issue	Specific manifestations	Future action directions	References
Adherence issues	Effective yet hard to apply	1. Adherence difficulties are rooted in structural constraints rather than a lack of personal motivation. 2. High work intensity, fragmented rest opportunities, and a lack of tailored support. 3. Emergency department staff exhibit marked reductions in energy, fluid, and macronutrient consumption during night shifts, alongside an increased prevalence of extended periods without food or fluid intake. 4. Interventions proven effective under strict trial conditions (e.g., 12-week light therapy) face real-world barriers including scheduling uncertainty, lifestyle variability, and limited organizational support.	1. Move beyond theoretically optimal models toward context-sensitive, implementable protocols. 2. Embed interventions within the real-world routines and personal circumstances of healthcare workers. 3. Develop interventions that are more closely aligned with real-world clinical settings.	(66, 100, 112)
Limited long-term data	Causal links and long-term effects unclear	1. Existing evidence is predominantly based on cross-sectional or short-term cohort studies. 2. It is difficult to capture the cumulative effects of night shift exposure or the carryover effects after retirement. 3. Retired night shift workers did not show a significant overall risk of metabolic syndrome, but sex-specific metabolic abnormalities were observed. 4. RCT intervention periods are short (weeks to months), making it impossible to assess long-term effects on cardiovascular events or mortality.	1. Establish longitudinal cohorts covering the full occupational lifecycle (e.g., EPHOR-NIGHT, 1,001 Nights). 2. Integrate digital monitoring technologies, including wearable devices and biomarkers. 3. Collect data from post-retirement populations to clarify the dynamic evolution of legacy effects and their potential reversibility.	(39, 46, 66, 95, 113, 114)
Poor cross-disciplinary teamwork	Complex mechanisms; translation remains a major challenge	1. Encompasses diverse domains, including circadian biology, neuroendocrine regulation, immune-inflammatory pathways, metabolic control, and psychosocial determinants. 2. A persistent translational gap exists between mechanistic insights and the development of actionable intervention frameworks. 3. The synergistic effects of chrononutrition with light therapy, sleep optimization, and pharmacological interventions have not been systematically explored. 4. Shift scheduling optimization requires integration of chronobiology, human resource management, and employee preferences.	1. Establish an interdisciplinary collaboration network involving clinicians, basic scientists, epidemiologists, nutritionists, sleep specialists, and hospital administrators. 2. Adopt multidisciplinary, individualized comprehensive lifestyle interventions. 3. Jointly develop and validate a “one person, one strategy” precision health promotion system.	(48, 97, 115–117)

### 6.1 Adherence issues

Although light therapy, chrononutrition, and behavioral approaches have been found to benefit the cardiometabolic health of night-shift healthcare workers, adherence in practice remains a serious challenge. Importantly, low adherence cannot be attributed simply to personal motivation. It is more deeply rooted in the systemic and time-related constraints that characterize night shift work. Qualitative studies show that night shift workers are well aware that night work negatively affects their diet and health. Yet when it comes to joining nutrition or sleep programs, they face multiple real-world barriers. These include a fast-paced work environment, fragmented rest breaks, and limited personalized support (100). Taking emergency healthcare workers as an example, one study found that during night shifts, they consumed significantly less energy, fluids, and key nutrients. The proportion of staff who went long periods without eating or drinking also rose considerably. This highlights the real difficulty of maintaining regular meals and adequate hydration in demanding clinical settings (112). Furthermore, it should be noted that although some interventions have been proven effective under strictly controlled trial conditions, such as the improvement of blood pressure rhythm and glucose tolerance by 12 weeks of timed light therapy (66). However, maintaining these benefits in everyday work environments is challenging due to unpredictable shift schedules, variations in personal habits, and limited organizational backing. This suggests that adherence is not just a behavioral issue. It reflects a deeper mismatch between workplace structures, environmental conditions, and individual circumstances. Going forward, intervention strategies should not stop at proving efficacy. They must be designed with a practical understanding of healthcare workers' routines and real-life needs, shifting from idealized protocols to practical. Only then can interventions become both feasible and sustainable. The adherence challenges of precision scheduling arise largely from organizational-level structural constraints. Human resource shortages are a key issue. As noted above, these shortages make it difficult to implement chronotype-based scheduling in most healthcare institutions. Even when scheduling plans are developed, employee compliance is not guaranteed. Several factors play a role. These include compensation systems, performance evaluations, and perceptions of fairness. Therefore, improving adherence requires not only individual education and incentives, but also the establishment of institutional safeguards at the organizational level. These should be built on three pillars: management policy support, flexible shift-swapping mechanisms, and reasonable financial subsidies.

### 6.2 Limited longitudinal data

Current evidence on the effects of night shift work on cardiometabolic health is derived mainly from cross-sectional studies or short-term cohort observations. The scarcity of long-term prospective follow-up data makes it difficult to establish causal relationships or determine the durability of intervention effects. This limits our ability to accurately evaluate the cumulative impact of night shift exposure and its long-term health outcomes. On a positive note, several high-quality cohort studies (e.g., EPHOR-NIGHT (113), the 1,001 Nights cohort (95)) are now collecting multidimensional biomarker data and using wearable devices. Nevertheless, most studies still have relatively short follow-up periods. This limits their ability to capture the cumulative health effects of night shift exposure or to assess whether risks persist after retirement (39). This reflects a notable temporal gap within the current evidence framework. For example, although a comparative study of retired night shift and day workers suggested the presence of sex-specific metabolic risk residuals, the overall prevalence of metabolic syndrome did not differ significantly between groups after covariate adjustment (39). This observation reinforces the need for extended longitudinal surveillance. Moreover, existing randomized controlled trials have lasted only weeks to months (e.g., 12-week light therapy (66), behavioral therapy (114)). This means they cannot assess whether interventions reduce cardiovascular events or mortality over several years. Although linked analyses based on national health surveys and death registries have confirmed that shift work is associated with elevated all-cause and cardiovascular mortality (adjusted HRs of 1.28 and 1.61, respectively) (46), these studies have not evaluated the long-term benefits of specific interventions. Consequently, there is an urgent need to establish longitudinal cohorts covering the full occupational lifecycle, integrated with digital monitoring technologies, to systematically evaluate the long-term health consequences of night shift exposure and the sustained benefits of intervention strategies. At the same time, data collection should extend to retired populations to elucidate the dynamic trajectory of legacy effects and their potential reversibility.

### 6.3 Multidisciplinary collaboration

The impact of night shift work on cardiometabolic health involves multiple dimensions, including circadian biology, neuroendocrine regulation, immune-inflammatory responses, nutritional metabolism, and psychosocial factors. Given this complexity, a single-disciplinary perspective is insufficient to fully elucidate the mechanistic network or to develop effective integrated countermeasures. Existing studies have preliminarily revealed multidimensional pathophysiological pathways. These include central and peripheral clock desynchronization (48), autonomic dysfunction (115), chronic low-grade inflammation, and the potential role of the microbiota–gut–brain axis (116). Yet, there remains a notable gap between these mechanistic insights and their translation into real-world intervention strategies. Taking chrononutrition as an example, although interventions have shown time-dependent modulatory effects on postprandial lipids and insulin responses (97), their synergistic effects with light management, sleep optimization, and pharmacological applications have not yet been systematically explored. Similarly, optimizing shift scheduling at the organizational level depends on combining chronobiological principles, human resource allocation, and employee preferences. This requires close collaboration across multiple fields including medicine, public health, engineering, psychology, and management. Recent systematic reviews have also emphasized the need for multidisciplinary, individualized comprehensive lifestyle interventions to reduce cardiometabolic disease risk (117). Therefore, future research should prioritize the formation of interdisciplinary networks encompassing clinicians, translational scientists, epidemiologists, nutritionists, sleep medicine specialists, and healthcare administrators. Such collaborative platforms would be essential for the co-development and rigorous validation of individualized, precision-oriented health promotion frameworks.

## 7 Conclusions

The adverse cardiometabolic consequences of prolonged night shift exposure among healthcare workers are now well substantiated, with growing evidence that circadian misalignment acts as an independent risk factor. Yet, in a healthcare system that must operate around the clock, simply reducing night shifts is not a viable option. Thus, the core challenge in this field has shifted from identifying health hazards to achieving precision protection while acknowledging the necessity of night shift work. This transition requires systematic progress on three fronts. At the research level, it is essential to go beyond cross-sectional studies and build longitudinal, multicenter cohorts covering the full career span. These should collect data on chronotype, gene expression, metabolic traits, and daily behaviors. Machine learning can then be used to build personalized risk prediction models that offer practical guidance for interventions. At the intervention level, uniform approaches should be replaced with chronobiology-based combination strategies. These include circadian phenotype-based risk assessment and shift matching, coordinated timing of light exposure and meals, and schedule planning that minimizes circadian disruption (e.g., using forward rotation and limiting consecutive night shifts to 3 days or fewer). At the management and policy level, sleep health and metabolic monitoring should be incorporated into occupational health systems for healthcare workers. Dedicated funds should support mental health, exercise, and nutrition programs, and electronic health records should enable real-time monitoring of high-risk individuals.

In conclusion, responding to night shift-related cardiometabolic damage in the next phase requires a fundamental shift. We must move from simply describing health harms to taking proactive, data-informed, and personalized protective actions. This is not only an urgent academic priority for occupational medicine but also a practical cornerstone for the sustainability of the healthcare workforce and the resilience of the health system.

## Author's note

To ensure accurate cultural representation, the authors' names are presented in Pinyin followed by their original Chinese characters: Xuelan Ye (叶雪兰), Chao Huang (黄超), Jiaxin Zhou (周嘉欣), Zhibing Qiu (邱志兵), Jieying Wu (伍杰莹), Hongmei Wu (吴红梅), and Linfeng Mo (莫林烽).
